# Fractalkine is Involved in Lipopolysaccharide-Induced Podocyte Injury through the Wnt/β-Catenin Pathway in an Acute Kidney Injury Mouse Model

**DOI:** 10.1007/s10753-019-00988-1

**Published:** 2019-03-27

**Authors:** Soulixay Senouthai, Junjie Wang, Dongdong Fu, Yanwu You

**Affiliations:** Department of Nephrology, Affiliated Hospital of Youjiang Medical University for Nationalities, No.18 Zhongshan Road II, Baise, 533000 Guangxi Zhuang Autonomous Region China

**Keywords:** Podocytes, LPS, FKN, Wnt signaling, β-Catenin, AKI mouse model

## Abstract

Injury to podocytes leads to proteinuria, a hallmark of most glomerular diseases as well as being associated with the progression of kidney disease. Activation of the Wnt/β-catenin pathway is associated with the pathogenesis of podocyte dysfunction and can play a role in renal injury. Furthermore, the expression of fractalkine (FKN) induced by lipopolysaccharides (LPS) is also one of crucial inflammation factors closely related to renal tissue damage. The aim of this study is to explore the mechanism of LPS-induced FKN expression leading to podocyte injury and contribute to acute kidney injury (AKI) through regulation of Wnt/β-catenin pathway. An AKI model was established for *in vivo* experiments and blood was collected for serum BUN and Cr measurement, and histopathological features of the kidneys were studied by PASM and IHC staining. For *in vitro* experiments, a mouse podocyte cell line was stimulated with different concentrations of LPS for 24 and 48 h after which podocyte viability and apoptosis of cells were evaluated. The expression of podocyte-specific markers, FKN and Wnt/β-catenin pathway mRNA and protein was detected in mice and cells by using qRT-PCR and western blotting. LPS induced the expression of FKN and activation of the Wnt/β-catenin pathway, leading to a decrease of podocyte-specific proteins which resulted in poor renal pathology and dysfunction in the AKI mouse model. Moreover, LPS treatment significantly decreased cell viability and induced podocyte apoptosis in a dose-dependent manner that causes changes in the expression of podocyte-specific proteins through activation of FKN and the Wnt/β-catenin pathway. Thus, the expression of FKN and Wnt/β-catenin pathway by LPS is closely associated with podocyte damage or loss and could therefore account for progressive AKI. Our findings indicate that LPS induce podocyte injury and contribute to the pathogenesis of AKI by upregulating the expression of FKN and Wnt/β-catenin pathway.

## INTRODUCTION

Acute kidney injury (AKI) is one of a number of conditions that affect kidney structure and function which results in a sudden episode of kidney failure that can occur over a matter of hours or days. It is a common complication of critically ill patients that is independently associated with increased mortality. Glomerular disease is a related cause of AKI which results in acute inflammation of blood vessels and glomeruli and is often seen in severe cases of acute glomerulonephritis [1]. It has been reported that podocyte injury is a pivotal factor of glomerular diseases and, in particular, it is also associated with the occurrence of AKI [2, 3]. Podocytes are terminally differentiated cells in the Bowman’s capsule of the kidneys that wrap around the capillaries of the glomerulus. They are attached to the outside of the glomerular basement membrane (GBM), a structure which is very important for the filtration of proteins and other molecules from the blood.

Injuries to podocytes (necrosis [4], apoptosis [5], and altered autophagy [6]) are considered to be the major contributors to the development of glomerular disease [7, 8] as their loss causes proteinuria and is associated with progressive AKI [2]. Foot processes (FP, derived from major processes) form a characteristic interdigitating pattern with FP of neighboring podocytes and are found to be in between the filtration slits that are bridged by the slit diaphragm (SD). The SD is a highly specialized intercellular junction between the podocyte FP and is crucial in the formation of the filtration barrier in the renal glomeruli. Nephrin, podocin, CD2-associated protein (CD2AP), and synaptopodin (SYNPO) are proteins considered to be the critical components of the epithelial SD and FP, and these can help maintain the integrity of the podocyte for avoiding proteinuria [9–11]. Recently, some investigators have reported that the levels of the SD and FP proteins, nephrin, podocin, CD2AP, and SYNPO, decrease in glomerular diseases [12–15]. Furthermore, these authors have highlighted the association between the expression of nephrin, podocin, CD2AP, and SYNPO proteins and the development of glomerular disease [9, 16, 17].

Lipopolysaccharides (LPS), also known as lipoglycans and endotoxins, are derived from the outer membrane of gram-negative bacterial cell walls, are released from proliferating or dying bacteria, and these are recognized by a variety of host cells. After bacterial infections, LPS is one of the major factors that lead to cell and tissue injury and is an inducing virulence factor for the pathogenesis of inflammation-associated diseases [18, 19]. In the kidney, it has been reported that LPS could be the underlying cause of albuminuria and aggravate glomerulonephritis in MRL/lpr mice [20]. LPS can stimulate the expression of inflammatory cytokines and activation of the NF-κB pathway in the pathogenesis of renal disease caused by injury, infection, and autoimmune factors [21–23]. Moreover, LPS is also one of the most important factors that lead to AKI [24, 25].

Fractalkine (FKN/CX3CL1) is a unique member of the CX3C chemokine family and participates in inflammatory response in several biological systems [26, 27]. It is also implicated in progression of a variety of renal diseases (glomerular inflammation and endothelial injury) [28, 29]. Park et al. found that LPS can upregulate expression levels of FKN and may contribute to renal inflammation leading to chronic renal allograft rejection [30]. In our previous studies, we showed that LPS can induce the expression of FKN increased in HK-2 cells and MRL/lpr mice which can cause cell damage, leading to proteinuria, renal dysfunction, and severity of renal pathology [31]. Therefore, our previous studies indicated that FKN induced by LPS is one of the major factors that lead to renal cell and tissue damage.

In addition, recent research has also shown that upregulation of Wnt/β-catenin signaling expression plays an essential role in kidney disease and is associated with podocyte injury both *in vivo* and *in vitro* [32–34]. The Wnt/β-catenin signaling pathway is considered to be an important developmental signaling pathway implicated in organogenesis and disease development in multicellular organisms, and kidney is one of these organs. In the kidney, it can regulate cell proliferation, survival, and cell behavior in both embryos and adults. However, in the mature kidney, the Wnt signaling pathway appears to be silenced, but it can be re-activated upon renal injury. Wang et al. confirmed that AKI in patients are caused by the activation of the Wnt/β-catenin signaling pathway [35]. Furthermore, it has been reported that activation of the Wnt/β-catenin pathway can aggravate LPS-induced inflammation [36, 37]. In addition, modulation of the Wnt/β-catenin pathway plays an important role in suppressing LPS-induced inflammatory response .

In this study, we detected expression of FKN and activation of Wnt/β-catenin signaling pathway in podocytes induced by LPS *in vitro* and *in vivo* and examined whether the expression of FKN occurring through regulation of Wnt/β-catenin pathway leads to podocyte injury by reducing expression of podocyte-specific proteins and contributes to risk factors that affect the development of AKI. Thus, our findings may constitute a key step in the pathogenesis of podocyte injury and could be a novel target for therapeutic intervention of AKI.

## MATERIALS AND METHODS

### Reagents

LPS (L2880 for animals and L4391 for cell culture) and XAV939 (X3004), tankyrase inhibitor (with a disruptor activity toward Wnt/β-catenin signaling) were purchased from Sigma-Aldrich, St. Louis, USA. LPS was dissolved in phosphate-buffered saline (PBS, Solabio, Beijing). XAV939 used in this study had a purity of ≥ 98%, and it was dissolved in dimethyl sulfoxide (DMSO, Sigma-Aldrich). Anti-mouse CX3CL1/Fractalkine (anti-mCX3CL1, R & D systems, MAB571) was also dissolved in PBS.

### *In Vivo* Studies

#### Animals

Eight-week-old female BALB/c mice weighing 23 ± 2 g were bought from the Model Animal Research Center of Nanjing University (Nanjing, China). All mice were housed under specific pathogen-free (SPF) conditions at 22–25 °C and kept in an environment of 40–60% relative humidity in the Animal Research Institute of Youjiang Medical University for Nationalities. All procedures involving mice were approved by the Committee on the Ethics of Animal Experiments of Youjiang Medical University for Nationalities and were carried out in accordance with the National Institute of Health guidelines.

#### Experimental Protocol

Thirty-five female BALB/c mice at 13 weeks of age were randomly distributed into seven groups that received intraperitoneal injections as follows: (1) once-a-day injection for 1 week of normal saline, control mice; (2) once-a-day injection for 1 week of normal saline and a single injection of LPS (10 mg/kg, i.p.) 24 h prior to sacrifice as described previously [38], LPS-induced AKI mice; (3) once-a-day injection for 1 week of anti-mCX3CL1 (5.0 μg/mouse, i.p), anti-FKN mice; (4) once-a-day injections for 1 week of normal saline and four times injection of XAV-939 (2.5 mg/kg, i.p) for 24 h prior to sacrifice, XAV mice [39]; (5) once-a-day injection for 1 week of anti-mCX3CL1 and a single injection of LPS 24 h prior to sacrifice, LPS + anti-FKN mice; (6) once-a-day injection for 1 week of normal saline and four times injection of XAV-939, together with a single injection of LPS for 24 h prior to sacrifice, LPS + XAV mice; (7) once-a-day injection for 1 week of anti-mCX3CL1 and four times injection of XAV-939, together with a single injection of LPS for 24 h prior to sacrifice, LPS + anti-FKN + XAV mice. The experiment was terminated after 24 h of treatment, the mice were killed, and the blood was collected for serum BUN and creatinine measurements, and the kidneys were harvested for pathological, quantitative RT-PCR, and immunoblotting analysis.

#### Blood Biochemical Assessment

Blood serum levels of urea nitrogen (BUN) and serum creatinine (Cr) were determined at 14 weeks when all mice were subsequently sacrificed 24 h after the last injection of LPS or XAV939 as described previously [31]. Blood samples of mice were collected by retro-orbital puncture under anesthesia, and their kidneys were isolated individually.

#### Histopathological and Immunohistochemical Analysis

To determine the extent of renal damage and cellular infiltration, kidney pathology was analyzed using paraffin sections. After 1 week of treatment, the mice were killed and kidney samples were fixed with 10% formalin in 0.01 moL/L phosphate buffer (pH 7.2) overnight and embedded in paraffin. Sections (3–4 μm thickness) were stained with periodic acid-silver methenamine (PASM) according to standard procedures for subsequent examination under a light microscope. The examination of renal pathology was performed in a blinded fashion by a pathologist.

For immunohistochemistry (IHC), formalin-fixed and paraffin-embedded renal sections were prepared as described previously, then the slides were dewaxed and hydrated. After that, the sections were immersed in 3% methanol hydrogen peroxide for 20–30 min and were then incubated with normal goat serum for 20 min to block endogenous peroxidase. The sections were incubated with individual primary antibodies including anti-nephrin (ab58968, 7 μg/mL dilution), anti-FKN (ab25088, 1:250 dilution) (Abcam Ltd., Hongkong, China), podocin (20384–1-AP, 1:450 dilution), β-catenin (17565–1-AP, 1:200 dilution) (Proteintech Ltd., Hubei, China), and wnt-4 (sc-376,279, Santa Cruz Biotechnology, California, USA, 1:300 dilution) overnight at 4 °C. The next day, they were incubated with goat polyclonal secondary antibody for 20 min at 37 °C, and then incubated with streptavidin-HRP, and the signal was then developed with DAB Substrate Kit (zli-9018, ZSGB-Bio Co., Ltd., Beijing, china). To compare the expression levels of nephrin, podocin, wnt-4, and β-catenin in renal cells by IHC, staining intensity was evaluated semiquantitatively following the methods developed previously [40], and fluorescence intensity was scanned and quantified by Image-Pro Plus v 5.1 software (Media Cybernetics Co, Ltd., USA). An intensity score was calculated as: % number of positive cells × values of staining intensity (from 0 to 3^+^) with the values typically ranging from 0 to a maximum of 300.

### *In Vitro* Studies

#### Cell Culture and Treatment

Mouse podocyte clone 5 (MPC5) cell line was obtained from the Cell Center of Fudan University (Shanghai, China) and cultured in Roswell Park Memorial Institute (RPMI) 1640 medium (Gibco) with 10% fetal bovine serum (Gibco) in T75 at 37 °C in a humidified 5% CO_2_ incubator with a daily change of medium. After 24-h incubation, podocytes were washed with sterile PBS buffer, and then 0.25% Trypsin-EDTA (1×) (Gibco) was added to the cells at room temperature. After that, cells were collected by centrifugation at 1000 rpm for 5 min and diluted (1:2) with the fresh medium for the generation of the preparation [41].

Podocytes were treated with different concentrations of LPS (0.01, 0.1, 1, and 10 μg/mL) at 24 h and 48 h, and then the viability of the podocytes were evaluated with a Cell Counting Kit-8 (CCK-8 assay). Replacement of LPS containing media after 24 h had no significant effect on cell viability, while replacement at 48 h resulted in a decrease of cell viability (*p* < 0.05). Cells were treated with those concentrations of LPS at 48 h for the detection of podocyte apoptosis by flow cytometry. Subsequently, cells were incubated with and without LPS (1 μg/mL), anti-mCX3CL1 (1.5 μg), and XAV939 (10 μM) [42] as required.

#### Cell Viability Assay

The effects of LPS treatment on podocyte viability were evaluated with a cell counting kit-8 (CCK-8, Solarbio) colorimetric assay. Briefly, podocytes were seeded at a density of 2 × 10^3^ cells/well (in 100 μL culture medium) in a 96-well plate. After LPS treatment, CCK-8 was added to each well, followed by a 2-h incubation at 37 °C in a 5% CO_2_ incubator. The absorbance was measured by the multimode microplate reader TriStar LB 941 (Berthold technologies, Bad Wildbad, Germany) at 450 nm.

#### Cells Apoptosis Analysis

Cells apoptosis was detected by the fluorescein isothiocyanate (FITC)-Annexin V/propidium iodide (PI) apoptosis kit (FITC-Annexin V/PI, BD Biosciences, San Diego, USA). Podocytes were seeded in 6-well plates, cultured at 37 °C in 5% CO_2_. After treatment for 24 h, cells were removed with 0.25% trypsin-EDTA. Cells were harvested by centrifugation at 300×*g* for 5 min at 4 °C. Cells were washed twice with cold PBS and then resuspended in 1× binding buffer at a concentration of 1 × 10^6^ cells/mL and incubated by 5 μL FITC-Annexin V and 5 μL PI at room temperature away from light for 15 min according to the manufacturer’s instructions. After that, podocyte apoptosis was determined by flow cytometry FACSCantoII (BD Biosciences, San Jose, CA, USA) within 1 h.

#### Quantitative RT-PCR Assays

Total RNA from whole kidney tissues or cells was extracted using TRIzol reagent (Invitrogen), according to the manufacturer’s instructions. For cDNA synthesis, reverse transcription was performed from total RNA (2 μg) by using the FastKing RT Kit (KR116, Tiangen, Beijing, China). The expression levels of nephrin, podocin, CD2AP, SYNPO, FKN, wnt-4, β-catenin, cyclin-D1, c-myc, and GAPDH were determined using SuperReal PreMix Plus (SYBR Green, FP205, Tiangen, Beijing, China) based on the manufacturer’s instructions. The primer sequences are shown in Table 1. The comparative gene expression was calculated by 2^−△△^Ct method as described previously [43].

**Table 1:** PCR Primers Used in This Study

Gene Name	(5′–3′)	Reverse (5′–3′)
Nephrin	TCCTGCTGCGATGGTGGTTG	GTCTGGGTTGCCTCCGATGG
Podocin	TGAGGATGGCGGCTGAGAT	GGTTTGGAGGAACTTGGGT
CD2AP	AGGAATTCAGCCACATCCACA	CGATCAATTCCAGTTCGTCCTC
SYNPO	GCTCGAATTCCGATGCAAATAAAC	CAGGCCACAGTGAGATGTGAAGA
Wnt-4	AAGAGGAGACGTGCGAGAAA	CACCACCTTCCCAAAGACAG
β-catenin	TTGCTGCTGGTTGGTTGGAAGG	CCAAGACATCTCGCAGTGAACTCC
Cyclin D1	CGTATCTTACTTCAAGTGCGTG	ATGGTCTCCTTCATCTTAGAGG
C-myc	AAATCCTGTACCTCGTCCGATT	CCACAGACACCACATCAATTTC
GAPDH	TGCTGAGTATGTCGTGGAGTC	GGAGATGATGACCCTTTTGG

#### Western Blot Analysis

After treatment of cells or kidney tissues for 24 h, total proteins were extracted from cells with RIPA buffer (Beyotime Biotechnology, Shanghai, China) containing a protease inhibitor cocktail (Cwbiotech, Beijing, China) (1:99). Protein samples were quantified, loaded, and separated by 8% sodium dodecylsulfate polyacrylamide gel electrophoresis (SDS-PAGE) and then transferred to a PVDF membrane (GE Healthcare, Freiburg, Germany). After transfer, 5% BSA (in TBST buffer) was used to block the membranes at room temperature for 1 h. Then, the membranes were pre-incubated with different primary antibodies including anti-nephrin, anti-FKN (Abcam, Hongkong, China), podocin, CD2AP, SYNPO, β-catenin (Proteintech, Hubei, china), wnt-4, cyclin-D1 and c-myc (Santa Cruz Biotechology, California, USA), and GAPDH (Danvers, MA, USA) antibodies at 4 °C overnight. After washing with TBST three times, membranes were incubated with secondary antibody. For visualization of detected proteins, immunoblots were analyzed using an enhanced chemiluminescence (ECL, Millipore, Billerica, MA, USA) western blot detection kit and the peroxidase luminescence intensity was measured using the Universal Hood II Molecular Imager GEL System (Bio-Rad, USA).

#### Statistical Analysis

Data are presented as the mean ± standard deviation. Inter-group comparisons were analyzed by one-way analysis of variance (ANOVA) for parametric data, F-test for equality of variances, and Newman-Keuls test for heterogeneity of variance. All analyses were conducted with SPSS 20.0 software (SPSS Inc., Chicago, USA). A *p* < 0.05 value was considered significant. Each experiment was repeated at least three times.

## RESULTS

### Inhibition of FKN and Wnt/β-Catenin Pathway Alleviated LPS-Induced Renal Histological Injury and Function Defects in AKI Mice

**Fig. 1:**
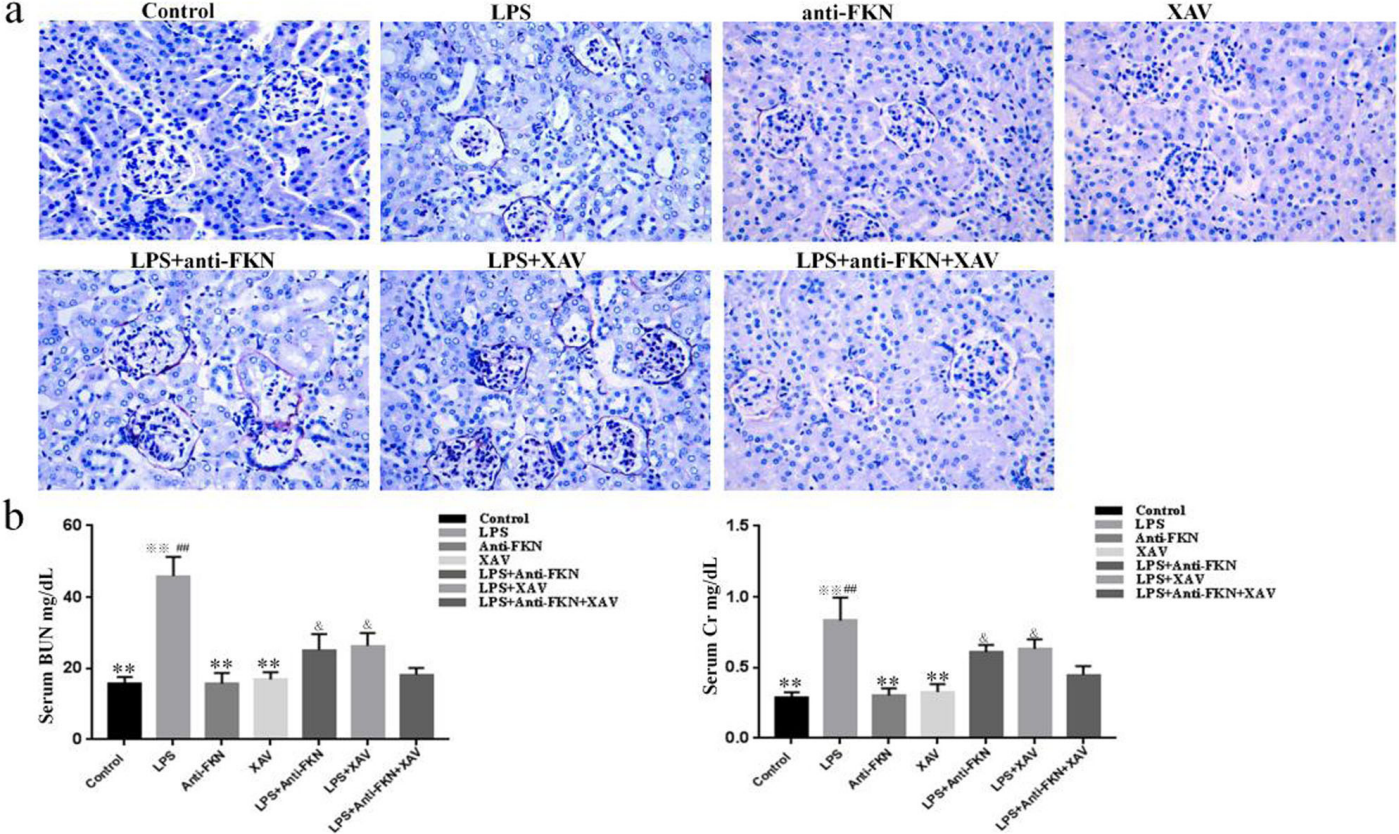
Effect of LPS-induced renal function defects and structural damage in the AKI mouse model. After all the injections have finished, the mice were sacrificed. Renal tissues were studied by PASM, and the blood was collected for serum BUN and creatinine measurements. **a** Histological findings in renal tissue of mice from the seven experimental groups and **b** assessment of kidney function in mice. **p < 0.01 when compared with LPS-induced AKI mice; ^※※^*p* < 0.01 when compared with LPS + anti-FKN mice; ^##^*p* < 0.01 when compared with LPS + XAV mice; ^&^*p* < 0.05 when compared with LPS + anti-FKN + XAV mice.

After the treatment was finished, we found that AKI mice group developed diarrhea and became inactive and lethargic after LPS injection at 24 h, but these symptoms were not found in the other groups of animals. The PASM-stained renal tissues appeared to have normal renal glomeruli and tubules in control, anti-FKN, and XAV mice. In contrast, in LPS-induced AKI mice, histopathological changes in the renal tissues showed marked glomerular atrophy, edema of renal tubular epithelial cells, destruction of tubular structures, dilation of renal capsule cavity, and renal interstitial edema of epithelial cells. While LPS + anti-FKN and LPS + XAV mice showed significantly diminished LPS-induced glomerular atrophy and edema of tubular and interstitial cells, the LPS + anti-FKN + XAV mice showed significantly decreased injury of the kidneys, more than both of the former groups (Fig. 1a).

Moreover, serum BUN and Cr levels in LPS-induced AKI mice (45.80 ± 5.40 and 0.83 ± 0.16) were also significantly increased compared to control (15.67 ± 1.89 and 0.29 ± 0.03), anti-FKN (15.70 ± 2.96 and 0.30 ± 0.05), and XAV mice (16.92 ± 2.0 and 0.32 ± 0.05), respectively (*p* < 0.01). However, a significant decrease was seen in LPS-induced AKI mice when compared to LPS + anti-FKN (25.14 ± 4.52 and 0.61 ± 0.05) and LPS + XAV mice (26.38 ± 3.57 and 0.63 ± 0.06), respectively (*p* < 0.01). In LPS + anti-FKN + XAV mice (18.21 ± 1.93 and 0.44 ± 0.06), the decrease in serum BUN and Cr levels were more than in LPS + anti-FKN and LPS + XAV mice (*p* < 0.05) (Fig. 1b).

### Expression of Nephrin, Podocin, Wnt-4, β-Catenin, and FNK Protein in Renal Tissue of LPS-Induced AKI Mice

**Fig. 2:**
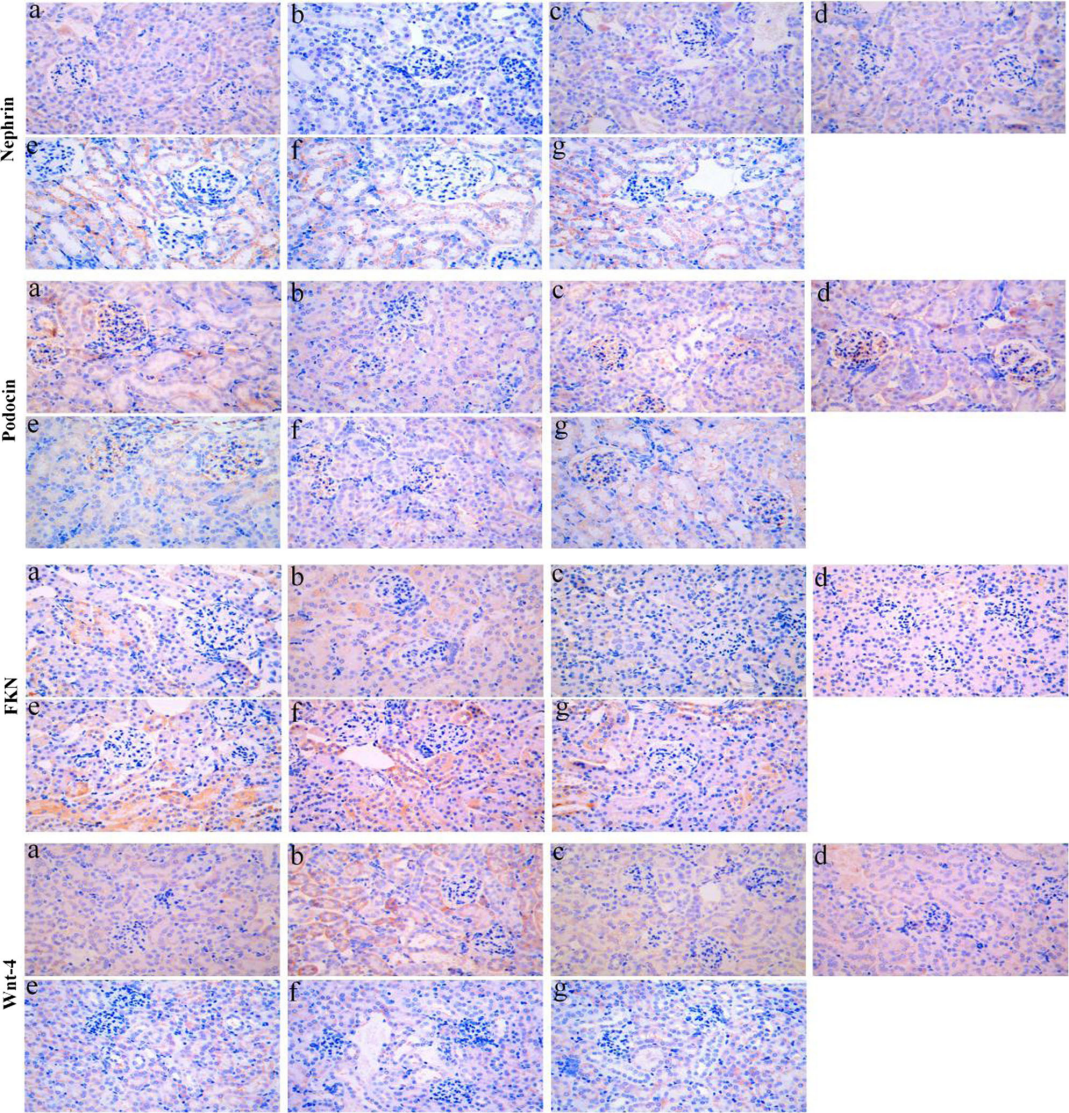

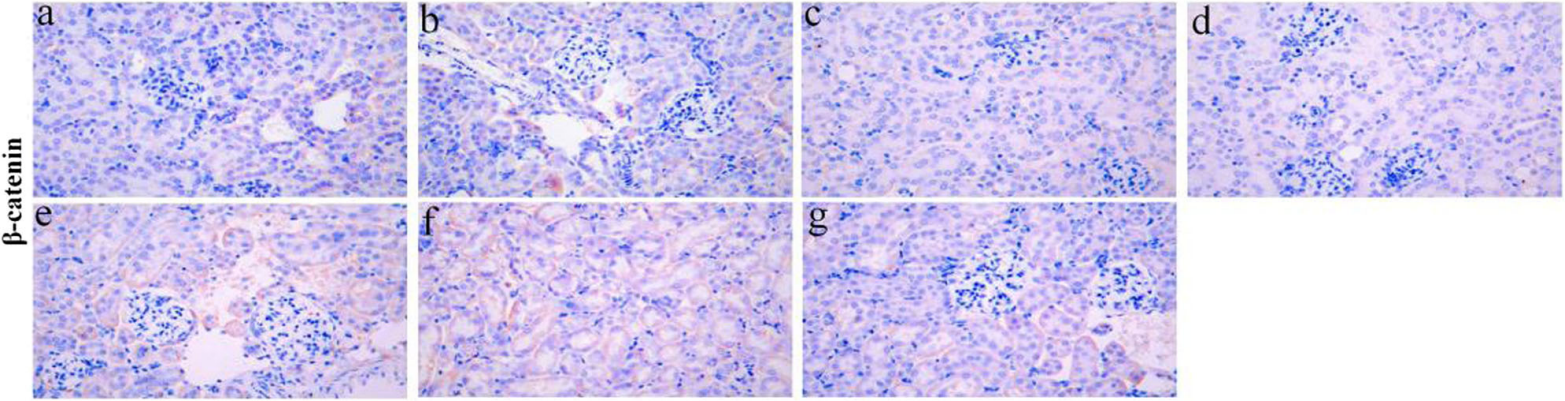
The expression of nephrin, podocin, FKN, wnt-4, and β-catenin protein in the kidneys of mice. Renal tissue sections of mice were stained by IHC (original magnification, × 400). **a** Nephrin and podocin; **b** FKN and wnt-4; **c** β-catenin. a Control, b LPS-induced, c anti-FKN, d XAV, e LPS + anti-FKN, f LPS + XAV, and g LPS + anti-FKN + XAV mice.

In kidney sections of AKI mice stained by IHC, the expression of nephrin, podocin, FNK, β-catenin, and wnt-4 proteins were compared between the different groups of mice (Fig. 2). According to semiquantitative evaluation, the intensity scores of nephrin were 120.6 ± 9.01, 12.0 ± 4.58, 104.3 ± 14.97, 110.3 ± 14.01, 67.6 ± 7.50, 64.0 ± 6.55, and 86.0 ± 6.0; podocin were 151.3 ± 16.28, 19.6 ± 4.50, 154.6 ± 11.71, 161.0 ± 9.53, 57.6 ± 7.09, 62.0 ± 7.21, and 96.0 ± 5.56; FKN were 36.3 ± 9.29, 141.0 ± 3.60, 20.3 ± 2.51, 36.33 ± 3.51, 100.6 ± 6.65, 107.3 ± 3.78, and 65.3 ± 7.23; wnt-4 were 35.6 ± 7.37, 166.0 ± 11.0, 35.6 ± 4.50, 25.0 ± 4.58, 127.3 ± 15.27, 106.3 ± 6.42, and 75.6 ± 10.96; and β-catenin were 11.0 ± 3.60, 94.0 ± 5.56, 9.3 ± 3.21, 6.3 ± 2.08, 61.6 ± 6.50, 50.6 ± 9.45, and 24.0 ± 5.29 in control, LPS-induced, anti-FKN, XAV, LPS + anti-FKN, LPS + XAV, and LPS + anti-FKN + XAV mice, respectively.

There was decreased expression of nephrin and podocin protein in LPS-induced mice when compared to control mice (*p* < 0.01 and *p* < 0.05 respectively) and increased expression in LPS + anti-FKN and LPS + XAV mice compared to LPS-induced (*p* < 0.01 and *p* < 0.05 respectively) (Fig. 2). While the expression of FKN, wnt-4, and β-catenin was increased in LPS-induced mice compared to control mice, there was a decrease in LPS + anti-FKN and LPS + XAV mice (Fig. 2). Moreover, in LPS + anti-FKN + XAV mice, there was increased nephrin and podocin and decreased FKN, wnt-4, and β-catenin protein expression when compared to LPS + anti-FKN and LPS + XAV mice (*p* < 0.01 and *p* < 0.05 respectively).

### Effect of LPS at Different Concentrations on Podocyte Cell Viability

**Fig. 3:**
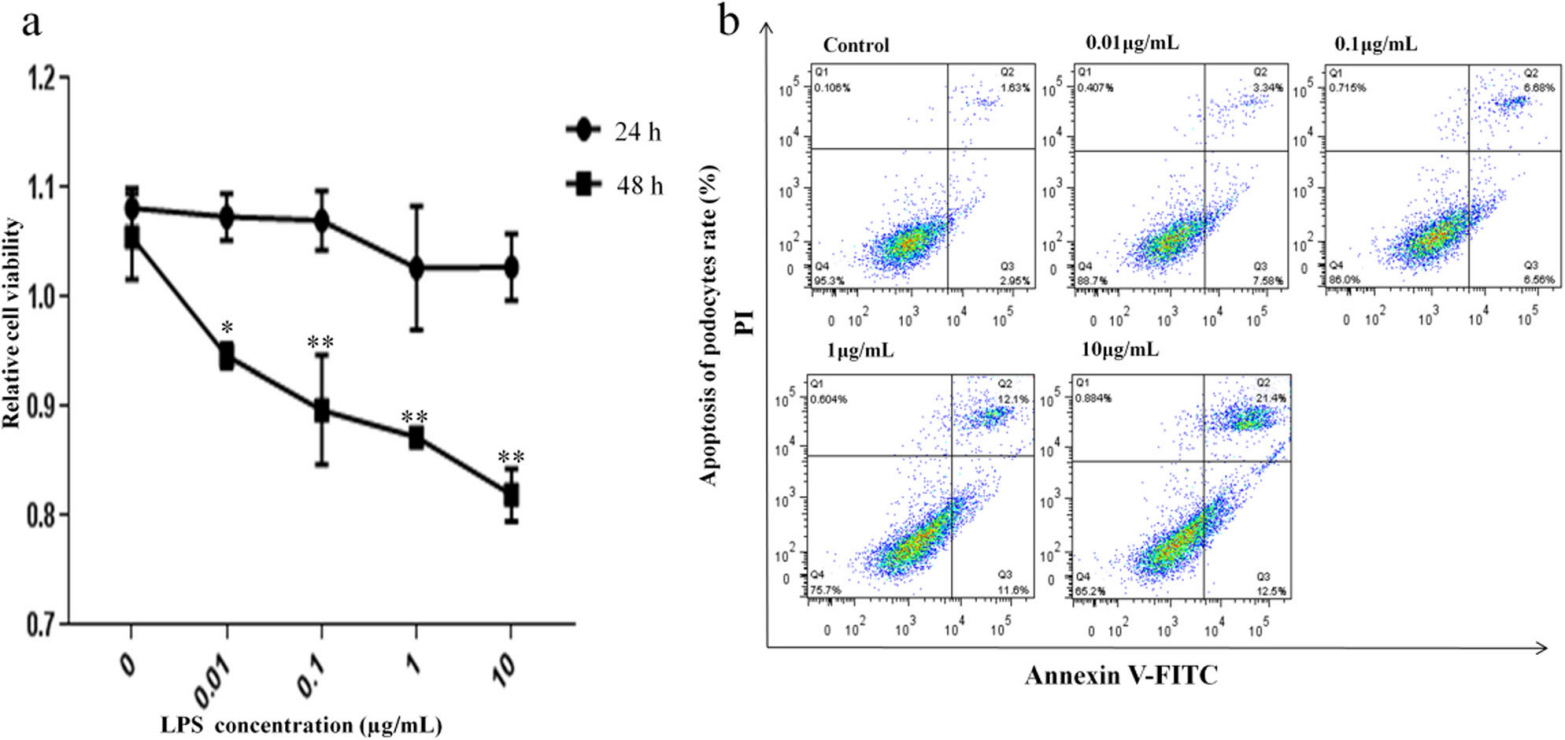
LPS inhibited podocyte viability and induced apoptosis in a dose-dependent manner. Cells were incubated with indicated concentrations (0.01, 0.1, 1, and 10 μg/mL) of LPS for 24 and 48 h. Cell growth inhibition activity of LPS was assessed by the CCK-8 assay and apoptosis was measured by Annexin V-FITC/PI staining and flow cytometry. **a** The viability of podocytes at different concentrations of LPS at 24 and 48 h and **b** ratio of apoptotic cells to the total number of cells induced by LPS at 48 h. The number of apoptotic cells equals the sum of the cells in the Q2 (early-stage cell apoptosis rate) and Q3 (late-stage cell apoptosis rate) regions. *p < 0.05; **p < 0.01 when compared with control group.

To determine whether LPS has cytotoxicity, it was noted that the viability of the podocytes had a tendency to decrease as the concentrations of LPS increased for incubations of 24 h and 48 h as seen by the CCK-8 assay. After 48 h of incubation with LPS at concentrations of 0.01, 0.1, 1, and 10 μg/mL, we observed a dose-dependent decrease in cell viability compared with controls (Fig. 3a). However, there was no significant difference between cells incubated with LPS for 24 h and controls. This shows that LPS decreases cell viability and induces a dose- and time-dependent cytotoxicity in these cells. Based on these results, we choose to treat cells for 48 h in subsequent studies.

### Different Concentrations of LPS-Induced Apoptosis of Podocytes

To determine whether the cytotoxic effect of LPS was due to induction of apoptosis, podocytes were treated with different concentrations of LPS for 48 h and then cell apoptosis was determined by Annexin V-FITC/PI staining and flow cytometry analysis. As shown in Fig. 3b, apoptosis rates of podocytes (% cells in zones Q2 and Q3 combined) were significantly increased after treatment with LPS from 15.6%, 24.4%, to 33% at 0.1, 1, and 10 μg/mL, respectively. The apoptosis rate in control cells was 5.4%. However, there was no significant difference between cells incubated with LPS at concentrations of 0.01 μg/mL (9.3%) and controls. This demonstrated that LPS treatment induced a dose-dependent apoptosis on cultured MPC5 cell line. Therefore, this study subsequently used 1 mg/mL LPS at 48 h for treatment of podocytes.

### Changes in Gene Expression of Podocytes, Chemokines, and Wnt/β-Catenin Signaling *In Vitro* and *In Vivo* Induced by LPS

**Fig. 4:**
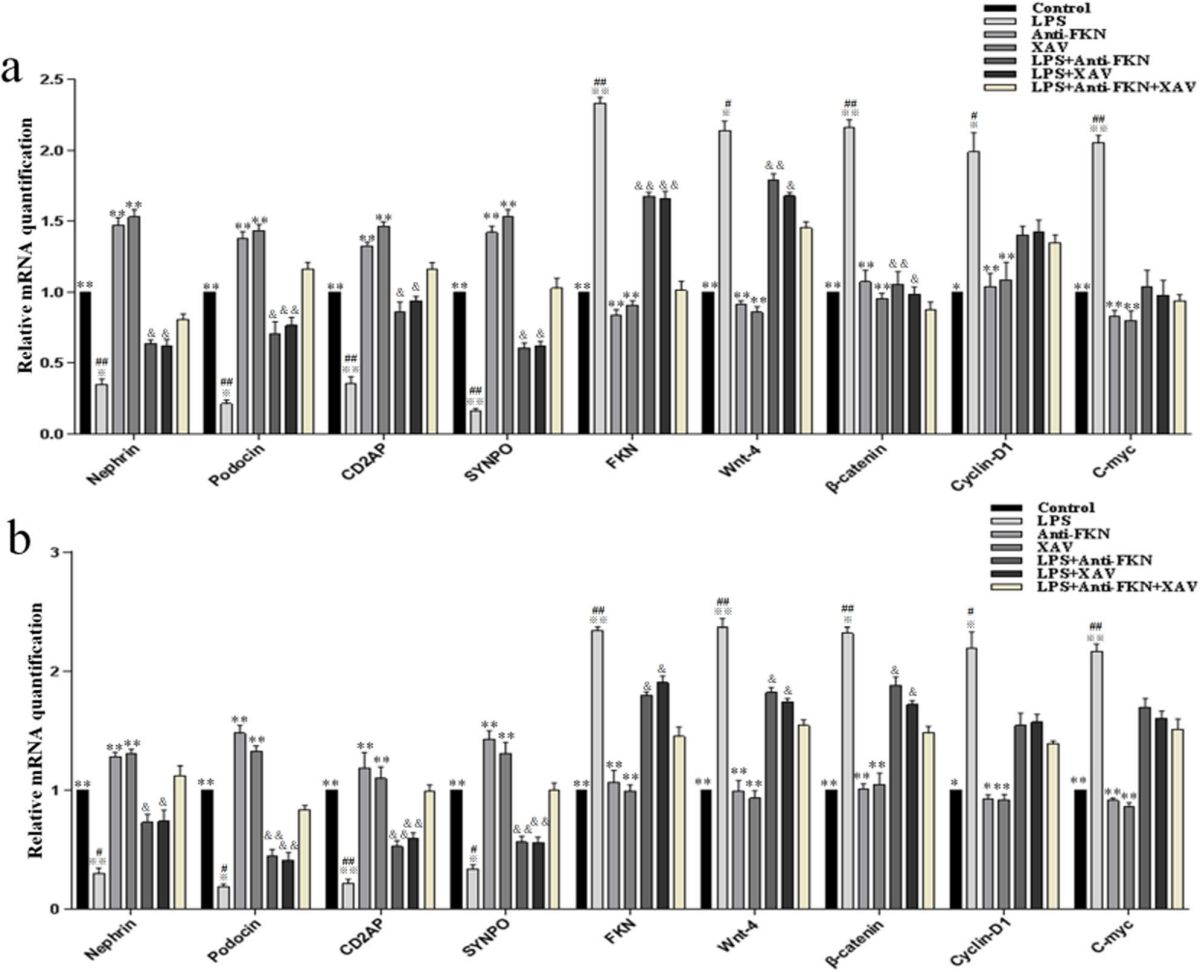
Expression of nephrin, podocin, CD2AP, SYNPO, FKN, wnt-4, β-catenin, cyclin-D1, and c-myc mRNA levels *in vitro* and *in vivo.* Total RNA was extracted from renal tissue of mice and podocytes. Then, the RNA was reverse-transcribed into complementary DNA (cDNA) and the transcripts were quantified using real-time PCR. The relative expression levels were determined by normalizing each target to GAPDH. **a** The expression of these proteins in podocytes and **b** the expression of these proteins in mice. *p < 0.05; **p < 0.01 when compared with LPS-induced AKI mice; ^※^*p* < 0.05; ^※※^*p* < 0.01 when compared with LPS + anti-FKN mice; ^#^*p* < 0.05; ^##^*p* < 0.01 when compared with LPS + XAV mice; ^&^*p* < 0.05; ^&&^*p* < 0.01 when compared with LPS + anti-FKN + XAV mice.

To assess the effects of LPS on renal injury *in vitro* and *in vivo*, the levels of nephrin, podocin, CD2AP, SYNPO, FKN, wnt-4, β-catenin, cyclin-D1, and c-myc mRNA were examined by qRT-PCR. As shown in Fig. 4, the results show that there was decreased expression of nephrin, podocin, CD2AP, and SYNPO mRNA and increased expression of FKN, wnt-4, β-catenin, cyclin-D1, and c-myc mRNA *in vitro* and *in vivo* on LPS-induced groups compared to control, anti-FKN, and XAV groups (*p* < 0.01 and *p* < 0.05). While LPS + anti-FKN and LPS + XAV groups *in vitro* and *in vivo* showed significantly higher expression of nephrin, podocin, CD2AP, and SYNPO and lower expression of FKN, wnt-4, β-catenin, cyclin-D1, and c-myc mRNA when compared to in LPS-induced group (*p* < 0.01 and *p* < 0.05). Also, the LPS + anti-FKN + XAV group had higher expression of nephrin, podocin, CD2AP, and SYNPO mRNA and lower expression of FKN, wnt-4, and β-catenin mRNA when compared to the LPS + anti-FKN and LPS + XAV groups (*p* < 0.01 and *p* < 0.05).

### Inhibition of FKN Expression Elevated the Protein Levels of Podocytes and Restrained the Wnt/β-Catenin Pathway *In Vitro* and *In Vivo* by LPS-Induced

**Fig. 5:**
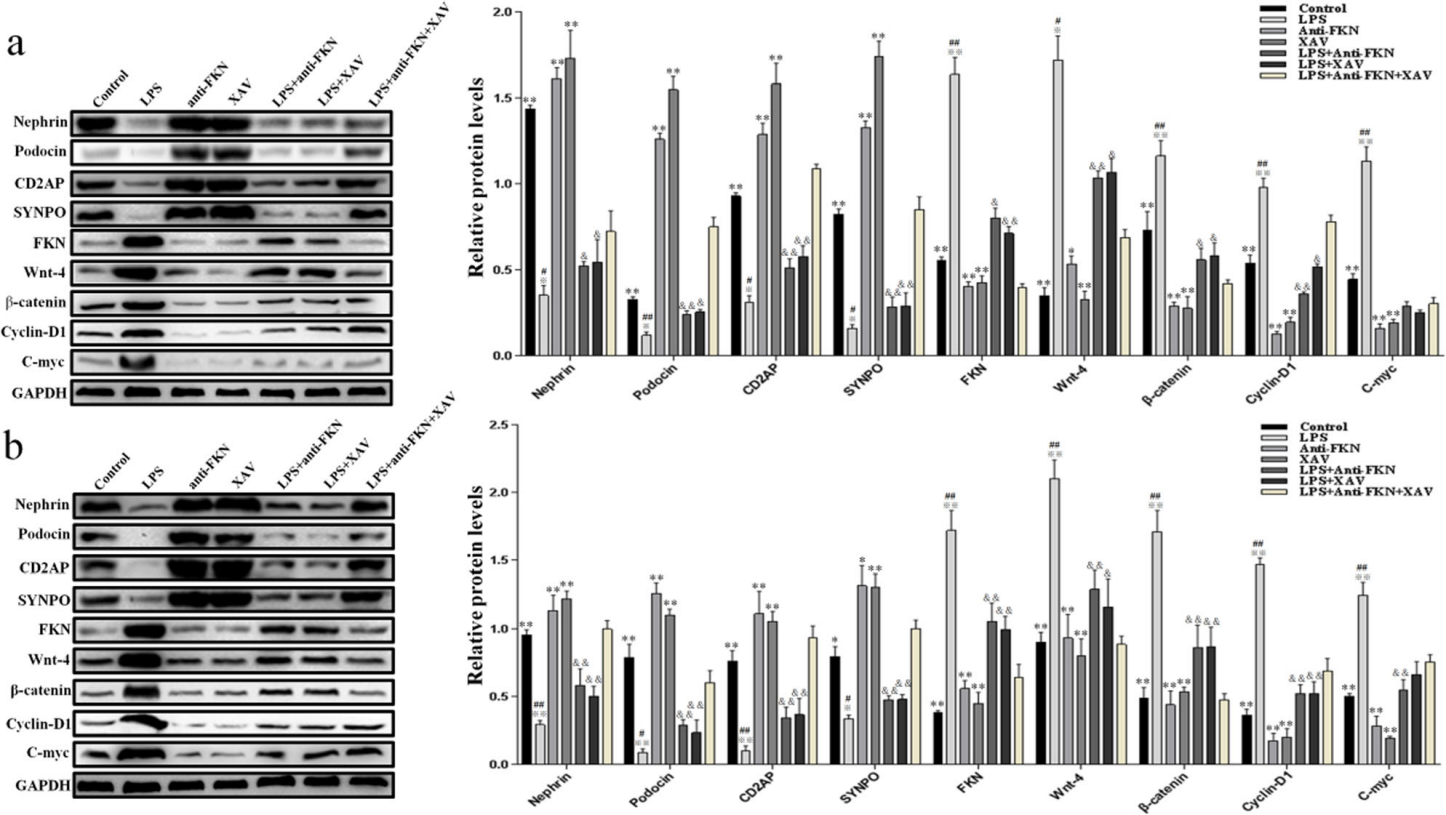
Expression of nephrin, podocin, CD2AP, SYNPO, FKN, wnt-4, β-catenin, cyclin-D1, and c-myc protein levels *in vitro* and *in vivo*. The expression of these protein in podocytes and renal tissue induced by LPS was evaluated using western blotting analysis. **a** The expression of these proteins in podocytes and **b** the expression of these proteins in mice. *p < 0.05, **p < 0.01 when compared with LPS-induced AKI mice; ^※^*p* < 0.05; ^※※^*p* < 0.01 when compared with LPS + anti-FKN mice; ^#^*p* < 0.05; ^##^*p* < 0.01 when compared with LPS + XAV mice; ^&^*p* < 0.05; ^&&^*p* < 0.01 when compared with LPS + anti-FKN + XAV mice.

The expression levels of nephrin, podocin, CD2AP, SYNPO, FKN, wnt-4, β-catenin, cyclin-D1, and c-myc proteins *in vitro* and *in vivo* of the kidneys were analyzed by western blot. As shown in Fig. 5, the results indicated that LPS inhibited the expression of podocyte-specific proteins, while it stimulated the expression of FKN and the Wnt/β-catenin pathway. The LPS-induced group showed significant decreases in expression levels of nephrin, podocin, CD2AP, and SYNPO proteins while increases in expression levels of FKN, wnt-4, β-catenin, cyclin-D1, and c-myc proteins when compared to control, anti-FKN, and XAV groups both *in vitro* and *in vivo* (*p* < 0.01 and *p* < 0.05). However, there was an increased expression of nephrin, podocin, CD2AP, and SYNPO proteins and a decreased expression of FKN, wnt-4, β-catenin, cyclin-D1, and c-myc mRNAs in LPS + anti-FKN and LPS + XAV groups when compared to the LPS-induced groups (*p* < 0.01 and *p* < 0.05). In the LPS + anti-FKN + XAV group, there was increased expression of nephrin, podocin, CD2AP, and SYNPO proteins and decreased expression of FKN, wnt-4, β-catenin, and cyclin-D1 proteins when compared to the LPS + anti-FKN and LPS + XAV groups (*p* < 0.01). These results are consistent with the changes seen in mRNA expression and to the IHC findings.

## DISCUSSION

The present study revealed that LPS can cause inflammatory responses both *in vitro* and *in vivo* of glomerular podocytes by means of enhancing FKN, wnt-4, β-catenin, cyclin-D1, and c-myc mRNA and protein synthesis. Moreover, the signal-transducing mechanisms of LPS-induction regulate FKN expression, leading to activation of Wnt/β-catenin pathway. These findings indicate that LPS may induce FKN synthesis and activate the Wnt/β-catenin signaling pathway in podocytes, resulting in a decrease in the expression of podocyte-specific mRNA and proteins and is involved in the occurrence and development of AKI.

A reduction in podocyte number after an injury or through apoptosis induced by external factors or drugs is a hallmark in the development of glomerulopathies [44, 45]. Furthermore, the inciting injury to the podocyte may vary among these glomerular diseases and the inevitable consequence of podocyte injury is loss of SD proteins, actin cytoskeleton derangement, and loss of structural integrity, leading to eventual FP effacement and podocyte apoptosis. These SD- and FP-specific proteins, especially nephrin, podocin, CD2AP, and SYNPO are crucial in signal transduction regulating a number of cell processes such as cell polarity, cytoskeleton organization, and survival of podocytes. Recent studies have demonstrated that a disruption of the FP and SD can lead to loss of their main protein components: nephrin, podocin, CD2AP, and SYNPO [46, 47].

Nephrin is synthesized by glomerular podocytes and is localized at the SD area between the podocyte FP, and it helps to maintain the interaction between the basement membrane and the podocytes of the epithelial cells. While podocin interacts with the cytosolic tail of nephrin. CD2AP serves as an adaptor protein in the structural organization of the SD and participates in a common signaling pathway necessary to maintain crucial podocyte functions [48]. SYNPO is a podocyte FP-specific actin-binding protein, which plays a crucial role in actin-based cell shape and motility and is also critical for stabilizing SD integrity because it is bound to nephrin through its direct interaction with CD2AP [49]. Thus, these proteins play major roles in maintaining the structural and functional integrity of the GBM and reduced expression of these proteins may contribute to the development of glomerular disease and lead to the pathogenesis of AKI.

Recently, studies demonstrated that LPS has the ability to cause a robust change in transcriptional activity of NF-κB and to increase the expression of IL-6, IL-8, IL-1β, and TNF-α markedly, leading to cell apoptosis. It has also has been reported that alterations in NF-κB activity might contribute to podocyte disorders in idiopathic nephrotic syndrome. According to a recent report, LPS is able to inhibit podocyte autophagy, which contribute to LPS-induced injury of podocytes. Tarak et al. reported that LPS can deform morphology and disrupt the actin cytoskeleton of podocytes and induce it proceed to injury [50]. Furthermore, LPS is recognized as the cause of kidney injury and it is regarded to be linked to the mechanisms of pathogenesis of AKI in mice [38, 51]. Therefore, LPS is one of the causes of podocyte injury and is a critical determinant in the pathogenesis and progression of AKI. The results from this study indicate that LPS inhibited podocyte proliferation and induced their apoptosis by regulating the expression of nephrin, podocin, CD2AP, and SYNPO levels and led to the injury of podocytes, which plays an important role in the development and progression of AKI. Most importantly, research has also found that LPS-induced typical changes in podocytes by upregulating the expression of FKN and activating the Wnt/β-catenin pathway.

FKN is a chemokine that plays an important role in modulating inflammation in the kidneys. Studies have shown that in humans and rodents, FKN expression is prominent in renal diseases, particularly in glomerular inflammation and endothelial injury [52, 53]. Zhuang et al. [54] found that FKN was produced and increasingly expressed in various cells of kidney (such as podocytes, mesangial cells, endothelium, and tubular epithelium) when stimulated by inflammatory factors. It has been revealed that LPS stimulation increases FKN expression through the activation of the NF-κB signaling pathway *in vitro* [55]. In our previous studies, we showed that both *in vitro* and *in vivo*, LPS can induce the expression of FKN and this can lead to inflammation of cells and tissues, which in turn causes proteinuria, renal dysfunction, and structural damage. Our data demonstrate that LPS induced FKN expression and led to a decrease of nephrin, podocin, CD2AP, and SYNPO levels, resulting in podocyte damage or loss, and this was a causal factor in the pathogenesis of AKI. Moreover, FKN is critically involved with the Wnt/β-catenin pathway in inducing podocyte injury and promoting the progression of kidney damage in AKI.

Previous studies proved overexpression of CX3CR1, the receptor for FKN, is closely related to the pathogenesis of osteoarthrosis by regulation of chondrocyte proliferation and apoptosis *via* the Wnt/β-catenin pathway [56]. In the spine, inhibition of the Wnt pathway could reduce FKN expression in remifentanil-induced post-operative hyperalgesia [57]. In HUVECs, regulation of the Wnt pathway plays an important role in inhibiting the expression of FKN in anti-inflammatory conditions induced by high glucose [58]. In the kidney, upregulation of the Wnt/β-catenin pathway is associated with podocyte injury and it can be involved in the pathogenesis of AKI. Furthermore, inhibition of the Wnt pathway plays an important role in suppressing the LPS-induced inflammatory response [59]. Consequently, the Wnt/β-catenin pathway is closely related to the expression of FKN induced by LPS. These results suggest that the expression of FKN and Wnt/β-catenin pathways was significantly increased *in vitro* and *in vivo* after LPS, which can lead to podocyte injury and resulting in the occurrence of AKI.

In summary, the present study suggests that nephrin, podocin, CD2AP, and SYNPO form a signaling complex that is important for supporting the functional and structural integrity of glomerular podocytes. In addition, LPS is one of the major factors that reduces expression of these podocyte-specific markers thereby mediating podocyte apoptosis and plays an important role in the occurrence and development of AKI by means of enhancing FKN and Wnt/β-catenin signaling pathway expression. This report may help shed light on one of causes of podocyte injury that contributes to the pathogenesis of AKI by upregulating the expression of FKN and Wnt/β-catenin pathway. However, we currently lack a full comprehensive understanding of the factors implicated in upregulating the expression of FKN through regulation of Wnt/β-catenin pathway. Further studies are required to determine the mechanisms and relationship between of FKN and the Wnt/β-catenin pathway, and how they are regulated in podocytes during AKI. This may potentially present new opportunities for the treatment and management of podocyte injury in AKI.

## Abbreviations

LPS: Lipopolysaccharides
FKN: Fractalkine
AKI: Kidney injury
GBM: Glomerular basement membrane
CD2AP: CD2-associated protein
SYNPO: Synaptopodin
FP: Foot processes
SD: Slit diaphragm
